# miR-92a and integrin expression in fibrovascular membranes in proliferative diabetic retinopathy

**DOI:** 10.3389/fopht.2023.1116838

**Published:** 2023-02-27

**Authors:** Qianyi Luo, Amir R. Hajrasouliha, Ashay D. Bhatwadekar

**Affiliations:** Department of Ophthalmology, Eugene and Marilyn Glick Eye Institute, Indiana University, Indianapolis, IN, United States

**Keywords:** diabetic retinopathy, miRNA, integrin, inflammation, fibrovascular membrane (FVM)

## Abstract

Diabetic retinopathy (DR) is a leading cause of vision impairment. The proliferative form of DR (PDR) involves fibrovascular membrane (FVM) formation at the vitreoretinal interface. MicroRNAs (miRNAs) are a class of non-coding RNA molecules that play an important role in gene regulation; a single miRNA could regulate multiple genes. We previously reported that miR-92a, a suppressor of integrins α_5_ and α_v,_ was downregulated in DR. Considering the integrin’s role in FVM pathology and the potential involvement of miR-92a in DR, we asked a question whether miR-92a could play a critical role in FVM pathology. We collected the FVM and epiretinal membranes of individuals with PDR and macular pucker (control) undergoing pars plana vitrectomy. The frozen sections of membranes were stained for α_5_ and α_v_β_3_ integrins. The miR-92a levels were assessed using real-time quantitative PCR. The FVMs of individuals with PDR stained brighter for integrin subunits α_5_ and α_v_β_3_ compared to the epiretinal membranes of subjects with macular pucker. miR-92a levels were decreased in FVM subjects. In conclusion, our studies demonstrate that miR-92a decrease is associated with an increase in integrins α_5_ and α_v_β_3,_ thus contributing to the inflammatory milieu in PDR.

## 1 Introduction

Diabetic retinopathy (DR) is one of the most common complications of diabetes. DR is broadly classified into four categories, a mild, moderate, or severe non-proliferative DR (NPDR) associated with an increase in retinal permeability and capillary occlusion, and a proliferative DR (PDR) characterized by new, abnormal vessels and the formation of a fibrovascular membrane (FVM). The FVM formation occurs at the interface of the ischemic and nonischemic retina and the optic nerve (1). Regardless of the stage of DR, inflammatory processes, growth of extracellular matrix (ECM), and angiogenesis together constitute a pathologic hallmark of DR. In PDR, the inflammatory milieu mediated by an increase in ECM production, chemokines such as C-C motif chemokine ligand 2 (CCL2) and hyperglycemia together increase the susceptibility of FVM formation (2). The ECM, along with chemokines and local inflammation, plays a critical role in FVM formation. In addition, the FVMs are associated with the migration and proliferation of various cells, such as macrophages, monocytes, fibroblasts, vascular endothelial cells, and bone marrow-derived cells (3).

Integrins are a family of multi-functional cell-adhesion molecules associated with α and β subunits acting as obligatory heterodimers on the cell surface (4). Integrins, *via* binding to ligands such as ECM proteins fibronectin, vitronectin, laminin, collagen, bone matrix protein, thrombospondin, and von Willebrand factor, play a preliminary role in cell-to-cell and cell-to-extracellular interactions. (5) In the retina, integrins are closely involved with the internal limiting membrane (ILM), thus playing an essential role in cell-matrix interactions (6). The capillary basement membrane thickening in DR results in an increase in integrin expression (7, 8). The α_v_β_3_ integrins are expressed on both basal and luminal surfaces of endothelial cells and on actively proliferating endothelial cells (9, 10) and inflamed cells in DR (9). Activation of α_v_β_3_ integrin maintains macrophage inflammatory response processes (11). Previous studies demonstrate an increase in the levels of α_v_β_3_, α_v_β_5,_ and α_5_ integrins in the FVMs of individuals with PDR (12, 13)

MicroRNAs are highly conserved non-coding RNA molecules involved in regulating gene expression. Emerging studies suggest that miRNA regulatory mechanisms play an important role in the pathogenesis of DR. miRNA profiling of retinal endothelial cells from diabetic mice reveals increased expression of miRNAs involved in retinal inflammation (14). We previously reported that miR-92a is uniquely regulated in circulating angiogenic cells (CACs) of individuals with DR; interestingly, miR-92a has been reported to be downregulated in rodent retina with diabetes (14). Previous studies show that miR-92a targets the mRNA of integrin α_v_ and integrin α_5_ (15). However, miR-92a and integrin expression in FVMs is not explored. This study shows that FVMs of PDR individuals exhibit downregulation of miR-92a with a concurrent increase in α_5_ and α_v_β_3,_ thus postulating a novel mechanism of a miR-92a-integrin axis in FVM formation in DR.

## 2 Materials and methods

### 2.1 Collection of FVM membranes and vitreous

We recruited individuals with active fibrovascular proliferation due to PDR and control individuals who are non-diabetic and operated to remove macular pucker during pars plana vitrectomy. The study was approved by the Institutional Review Board (IRB, # 1809532202), Indiana University School of Medicine, and conducted in accordance with The Code of Ethics of the World Medical Association. The informed consent was obtained before collecting the FVM membranes and vitreous. The membranes were placed immediately in ice-cold Hank’s balanced salt solution (Ca^++^ and Mg^++^ free), and vitreous samples were frozen separately. The FVMs were divided into pieces and processed for cryosectioning or total RNA isolation. The vitreous samples were also processed for RNA isolation.

### 2.2 miR-92a levels and integrin expression using qRT-PCR

The total RNA was isolated using a TRIzol reagent (ThermoFisher Scientific, Waltham, MA) per the manufacturer’s instructions. The purity of RNA was tested using NanoDrop 2000 UV-VIS spectrophotometer (ThermoFisher Scientific). Additional RNA purification rounds were performed using RNAEasy mini kit (Qiagen, Hilden, Germany), ensuring the 260/280 ratio of RNA as ~2. A 10 ng of RNA was reverse transcribed using the Applied Biosystems-TaqMan-MicroRNA reverse transcription kit (ThermoFisher Scientific), quantifying only a mature miRNA. miRNA-specific primer for miR-92a (hsa-miR92a-3P #000431, ThermoFisher Scientific) and housekeeping controls RNU6B (#001093 ThermoFisher Scientific) and RNU48 ((#00106, ThermoFisher Scientific) were used to quantify the levels of miR-92a in FVMs.

A 500 ng of RNA was reverse transcribed using SuperScript™ VILO cDNA Synthesis Kit (#11755-050 ThermoFisher Scientific). The following primers (ThermoFisher Scientific) were used *ITGAV* (#Hs00233808_m1), *ITGB3* (#Hs01001469_m1), *ITGA5* (#Hs01547673_m1), and a housekeeping control ACTB (#Hs99999903_m1). The qRT-PCR reaction was performed using ViiA7 real-time PCR system (ThermoFisher Scientific).

### 2.3 Integrin staining of FVMs

The FVM membranes were stored in Tissue-Tek OCT (Sakura FineTek-USA) compound prior to the cryosectioning. The sections of 8 μm thickness were obtained using Leica CM3050 Cryostat (Buffalo Grove, IL) followed by staining using integrin α_5_ (1:100, #CBL497, Millipore Burlington, MA) or α_v_β_3_ antibodies, (1:100, #MAB1976 Millipore). The CD31 antibodies (1:100, #ab28364 Abcam, Cambridge, MA) were used to stain endothelial cells. The following secondary antibodies were used, Alexa Fluor 555 goat anti-mouse (1:800 #A21422, ThermoFisher Scientific) or Alexa Fluor 488 goat anti-rabbit (1:800, #A11034ThermoFisher Scientific). The slides were mounted in Vectashield (Vector Laboratories, Burlingame, CA) mounting media containing DAPI. Three immunofluorescence images for each membrane were taken using Zeiss LSM-700 confocal microscope (White Plains, NY). The fluorescence intensity was quantified using ImageJ image processing software (16).

### 2.4 Statistics

The miR-92a expression values were expressed as 2^-dct^ with respect to individual housekeeping miRNA, RNU6B, and RNU48 levels. Individual FVM sample was run as three technical replicates and averaged. The final data in the chart were expressed as Mean ± SEM, where n corresponds respective FVM sample, biological replicates. The outliers were identified using Grubb’s method (Alpha 0.05). The statistical analysis of miR-92a expression was performed using the two-tailed Unpaired t-test using GraphPad Prism software (GraphPad Software, San Diego, CA, www.graphpad.com) version 9.4.1. The fluorescence intensity of retinal photomicrographs was analyzed using linear mixed models EM means followed by a comparison of the respective group with the least square design using IBM SPSS Statistics (New York, USA www.ibm.com/products/spss-statistics). The statistical significance was considered when the p-value was less than 0.05.

## 3 Results

### 3.1 A decrease in miR-92a in FVM membranes

**Figure 1:**
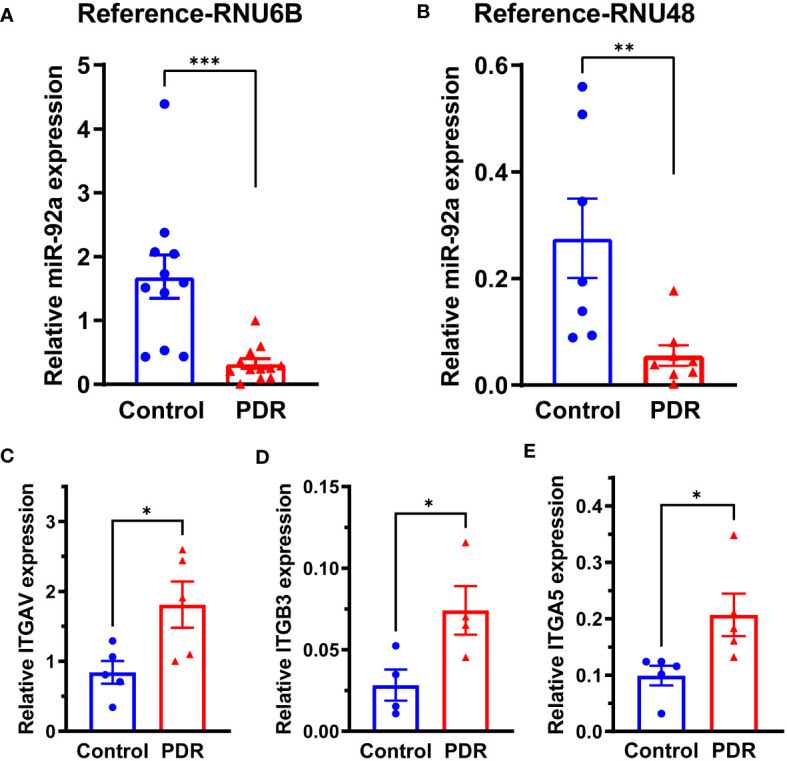
miR-92a decrease in FVM of individuals with PDR. Bar chart showing miR-92a levels in FVMs of individuals with PDR and epiretinal membranes of individuals with macular pucker using two separate housekeeping controls, **(A)** RNU6B (Control n=11, PDR n=12) and **(B)** RNU48 (Control n=7, PDR n=8). qRT-PCR was performed for mRNA targets of miR-92a, bar chart showing mRNA expression of **(C)** *ITAGV* (Control n=5, PDR n=5) **(D)** †*ITAGB3* (Control n=4, PDR n=4) **(E)** *ITAG5* (Control n=5, PDR n=5). n corresponds to biological replicate ***p<0.001, **p<0.01, *p<0.05, †outliers removed by Grubb’s method.

To study the miR-92a levels in FVM membranes, we obtained the FVM membranes from PDR individuals. The epiretinal membranes of non-diabetic patients undergoing surgery for macular pucker were used as a control. The miR-92a levels were decreased substantially for both housekeeping controls (RNU6B-p<0.001; RNU48-p<0.01) in the FVM of PDR subjects (**Figure 1**). While the miR-92a levels were decreased in vitreous samples of individuals with PDR, the difference was not statistically significant (**Supplemental Figure S1**).

### 3.2 An increase in αvβ3 and α5 integrins in FVM membrane

miR-92a is a known repressor of integrin α_5_, and α_v_ (15); to study whether miR-92a decrease in FVMs is coupled with an increase in integrin expression, we first determined mRNA expression of integrins ITGAV, ITGB3, ITGA5 that corresponds to integrin α_v_, β_3_ and α_5_ respectively. There was a 2.26-fold increase in ITGAV mRNA (**Figure 1C**; p<0.05), 3.5 fold increase in ITGB3 mRNA (**Figure 1D**; p<0.05), and a 2-fold increase in ITGA5 mRNA (**Figure 1E**; p<0.05).

**Figure 2:**
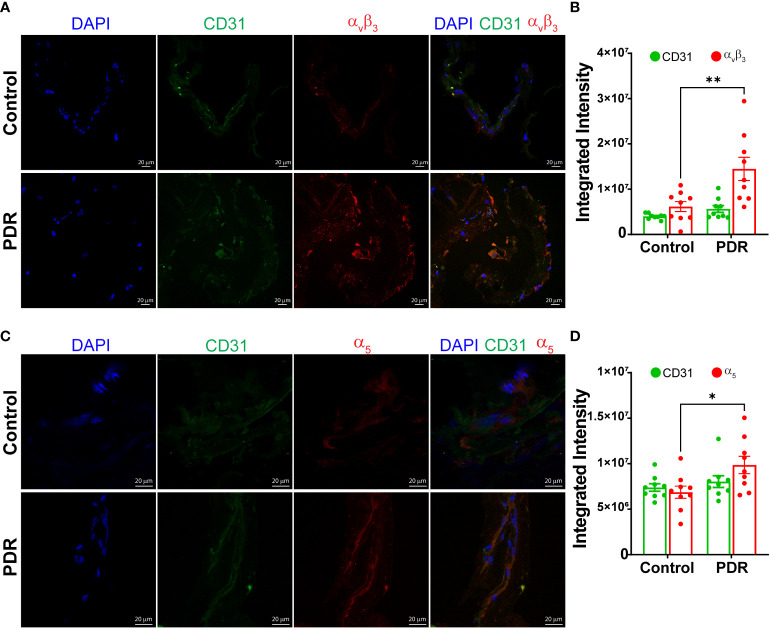
Increase in an integrin α_v_β_3_ and α_5_ expressions in FVM of PDR subjects. Representative photomicrographs showing staining and bar chart quantifying fluorescence intensity for α_v_β_3_ **(A, B)** and α_5_ **(C, D)** integrins in the FVM membranes of PDR subjects and control (macular pucker) individuals n=9 (3 images X 3 independent biological replicates). *p<0.05, **p<0.01.

Next, the epiretinal membranes were cryosectioned and stained for integrins α_v_β_3_ or α_5,_ The endothelial cells were labeled with CD31 antibodies. Both secondary antibody controls had minimal background staining (**Supplemental Figure S2**). The membranes of PDR subjects stained brighter for α_v_β_3_ antibodies compared to the control FVM membranes (**Figures 2A, B**). The α_5_ integrin staining exhibited a similar change in staining for PDR membranes; overall, the intensity of α_5_ staining was brighter for PDR-FVMs when compared to control epiretinal membranes (**Figures 2C, D**).

## 4 Discussion

FVM formation is the characteristic feature of PDR; our studies have identified a novel mechanism of FVM pathology by showing the involvement of the miR-92a-integrin axis. Our study’s salient finding includes that miR-92a decrease in FVM is associated with an increase in integrins α_5_ and α_v_β_3_, thus, postulating that miR-92a may be a critical anti-inflammatory target in the pathogenesis of PDR.

We report a decrease in miR-92a levels in FVMs for individuals with PDR; in line with our findings, previous studies demonstrate a reduction in miR-92a in vitreous of individuals with PDR (17) and the rodent retina with diabetes (14). While we observed a decrease in miR-92a in vitreous, this difference was insignificant. It is noteworthy that previous studies could not validate miR-92a in vitreous PDR (17–19) and plasma (19, 20) despite a decrease or increase in miR-92a levels in array platforms; this suggests that vitreous might not be an ideal tissue to study changes in miR-92a levels in context to DR, also severity of DR could have an effect on miR-92a levels.

While originally, the miR-17-92 cluster was found to be involved in tumorigenesis, emerging studies over the years have shown its important role in embryonic development, immune disease, cardiovascular diseases, aging, and neurodegenerative conditions (21). miR-17-92 cluster is highly expressed in endothelial cells; in particular, miR-92a has been shown to promote angiogenesis by targeting the mRNA of pro-angiogenic proteins, such as endothelial nitric oxide synthase (eNOS) and integrin α_5_ (15). miR-92a is reported to be activated in cancer cells and neuroblastoma, where it has acted as an endogenous repressor of angiogenesis (15). In our studies, we speculate that miR-92a decrease in FVM’s has played an integral role in aiding the inflammatory milieu of DR. The miR-92a reduction with a concurrent increase in integrin levels in DR might have promoted FVM formation in PDR individuals. There was no change in integrin expression and miR-92a levels in epiretinal membranes of subjects with macular pucker; however, we cannot rule out that macular pucker controls could exhibit changes in miR-92a levels, including a possible increase in miR-92a; this is one of the study’s limitations. Nonetheless, considering the present literature (14, 17), a decrease in miR-92a observed in PDR suggests a potential involvement of the diabetic microenvironment in mediating miR-92a decrease. miR-92a is reported as a negative regulator of inflammation in macrophages acting *via* MAP2K4, resulting in the downregulation of TLR4 and TLR2 (22). We confirmed this assertion in previous studies by showing that miR-92a overexpression in CACs decreases toll-like receptors (TLRs) and inflammatory cytokines such as TNF-α and IL1-β (23).

PDR is characterized by the formation of FVMs. Inflammatory processes, angiogenesis and growth of ECM components, such as fibronectin and collagen (24), constitute a pathologic hallmark of DR. Previous studies (12) and our findings demonstrate an increase in the levels of integrin α_v_β_3_ and α_5_ in fibrovascular membranes of individuals with PDR. While not establishing a direct link, we believe that miR-92a decrease had played an integral role in an increase in integrin expression in DR, considering its role as a negative regulator of these integrin molecules. We believe that optimum miR-92a levels may be necessary for reducing the severity of DR. Indeed, we previously reported that a cohort of individuals who remained free for DR maintained higher levels of miR-92a and a decrease in TLRs when compared to the subjects that developed DR. The reduction in TLR likely involves of c-Jun NH2-terminal kinases (JNK/c-Jun) pathway (22).

In conclusion, our studies have identified that the miR-92a decrease in FVM is associated with an increase in integrins α_5_ and α_v_β_3,_ which could contribute to the pathology of PDR. Furthermore, our studies pave the road for future therapeutic strategies involving miRNA therapeutics in treating DR individuals.

## Data availability statement

The raw data supporting the conclusions of this article will be made available by the authors, without undue reservation.

## Ethics statement

The studies involving human participants were reviewed and approved by Institutional Review Board, Indiana University School of Medicine. (IRB, # 1809532202). The patients/participants provided their written informed consent to participate in this study.

## Author contributions

Conceptualization, AB; methodology, QL; validation, QL; formal analysis, QL; resources, AB, AH; data curation, QL; writing—original draft preparation, AB; writing—review and editing, QL, AH, and AB; visualization, AB; supervision, AB; project administration, AB; funding acquisition, AB. All authors contributed to the article and approved the submitted version.
