# Supplementary material for: miR-92a and integrin expression in fibrovascular membranes in proliferative diabetic retinopathy
Source: Front Ophthalmol (Lausanne). Author manuscript; Available in PMC 2023 Jul 7. (PMC10327885; doi:10.3389/fopht.2023.1116838)
Supplement: Supplemental Figure 1 [file NIHMS1908929-supplement-Supplemental_Figure_1.pdf]

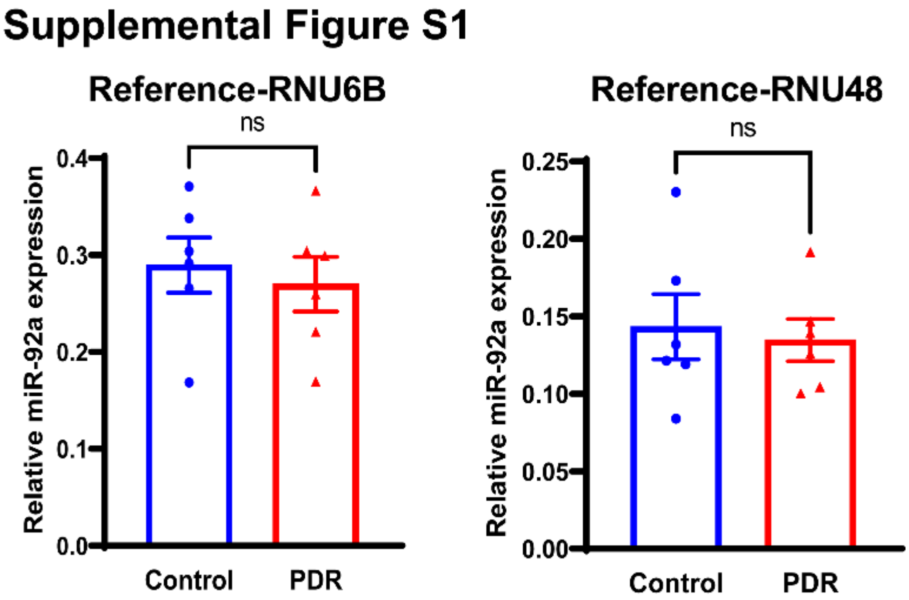

Supplemental Figure S1. miR-92a levels in vitreous of individuals with PDR. Bar chart showing miR-92a levels in vitreous of individuals with PDR (n=6) and epiretinal membranes of individuals with macular pucker (Control; n=6) using two separate housekeeping controls, RNU6B and RNU48; ns-not significant
