# Supplementary material for: miR-92a and integrin expression in fibrovascular membranes in proliferative diabetic retinopathy
Source: Front Ophthalmol (Lausanne). Author manuscript; Available in PMC 2023 Jul 7. (PMC10327885; doi:10.3389/fopht.2023.1116838)
Supplement: Supplemental Figure 2 [file NIHMS1908929-supplement-Supplemental_Figure_2.pdf]

Supplemental Figure S2

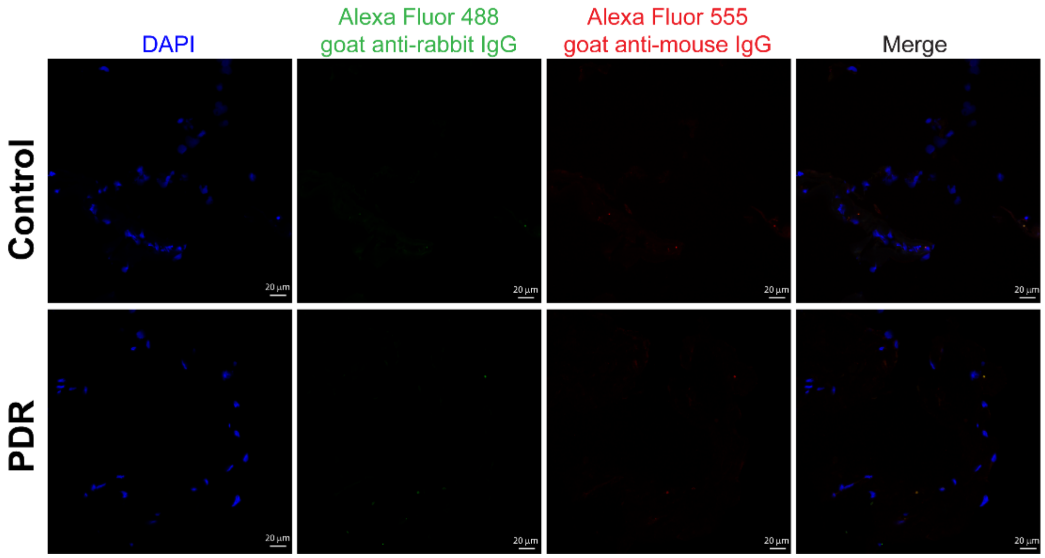

Supplemental Figure S2. Secondary antibody controls. Representative images show staining for secondary antibodies only.
